# Experimental and Machine-Learning-Assisted Design of Pharmaceutically Acceptable Deep Eutectic Solvents for the Solubility Improvement of Non-Selective COX Inhibitors Ibuprofen and Ketoprofen

**DOI:** 10.3390/molecules29102296

**Published:** 2024-05-14

**Authors:** Piotr Cysewski, Tomasz Jeliński, Maciej Przybyłek, Anna Mai, Julia Kułak

**Affiliations:** Department of Physical Chemistry, Pharmacy Faculty, Collegium Medicum of Bydgoszcz, Nicolaus Copernicus University in Toruń, Kurpińskiego 5, 85-096 Bydgoszcz, Poland; tomasz.jelinski@cm.umk.pl (T.J.); m.przybylek@cm.umk.pl (M.P.);

**Keywords:** non-selective COX inhibitors, ibuprofen, ketoprofen, deep eutectic solvents, solubility, machine learning, COSMO-RS

## Abstract

Deep eutectic solvents (DESs) are commonly used in pharmaceutical applications as excellent solubilizers of active substances. This study investigated the tuning of ibuprofen and ketoprofen solubility utilizing DESs containing choline chloride or betaine as hydrogen bond acceptors and various polyols (ethylene glycol, diethylene glycol, triethylene glycol, glycerol, 1,2-propanediol, 1,3-butanediol) as hydrogen bond donors. Experimental solubility data were collected for all DES systems. A machine learning model was developed using COSMO-RS molecular descriptors to predict solubility. All studied DESs exhibited a cosolvency effect, increasing drug solubility at modest concentrations of water. The model accurately predicted solubility for ibuprofen, ketoprofen, and related analogs (flurbiprofen, felbinac, phenylacetic acid, diphenylacetic acid). A machine learning approach utilizing COSMO-RS descriptors enables the rational design and solubility prediction of DES formulations for improved pharmaceutical applications.

## 1. Introduction

Ibuprofen (IP, dexibuprofen, CAS 51146-56-6) and ketoprofen (KP, dexketoprofen, CAS 22071-15-4) are nonsteroidal anti-inflammatory drugs (NSAIDs) [1,2] widely employed in clinical practice for their analgesic [3,4], antipyretic [5,6], and anti-inflammatory [7,8] properties. Chemically, both belong to the class of propionic acid derivatives. Ibuprofen, chemically known as (RS)-2-(4-(2-methylpropyl)phenyl)propanoic acid, and ketoprofen, 2-(3-benzoylphenyl)propionic acid, share structural similarities, differing primarily in their substituent groups. Their therapeutic efficacy stems from their ability to inhibit the cyclooxygenase (COX) enzymes, particularly COX-1 and COX-2, thereby impeding the synthesis of prostaglandins involved in inflammation, pain, and fever pathways [9,10,11,12]. The metabolism of ketoprofen and ibuprofen involves a series of enzymatic processes that primarily occur in the liver, leading to the formation of metabolites with varying pharmacological activities and elimination profiles. The two compounds undergo biotransformation primarily via cytochrome P450 (CYP) enzymes [13,14,15], and the resulting metabolites include hydroxylated forms, such as 2-hydroxyibuprofen, 3-hydroxyibuprofen, 3-hydroxyketoprofen, and 4-hydroxyketoprofen. The two drugs find extensive application in the management of various conditions, including musculoskeletal disorders [16,17], rheumatic diseases [18,19], and prolonged conditions such as Alzheimer’s [20], Parkinson’s [21], and Machado–Joseph diseases [22]. However, their usage is not devoid of hazards. Gastrointestinal complications, such as ulceration and bleeding, constitute notable adverse effects associated with both drugs, albeit with varying incidences [23,24,25]. Additionally, the prolonged administration of high doses of these NSAIDs may precipitate renal dysfunction [26] and cardiovascular events [27].

Solubility lies at the core of pharmaceutical science, exerting a profound influence on every stage of drug development and delivery [28,29,30]. Its importance resonates through the entire lifecycle of a medication, from initial formulation to therapeutic administration [31,32,33]. The solubility of a compound dictates its bioavailability, the rate and extent of absorption into the bloodstream, and consequently, its therapeutic efficacy [34,35]. Moreover, solubility profoundly impacts the stability of drug formulations, affecting shelf life and storage conditions [36,37]. Poor solubility poses significant challenges, necessitating higher doses to achieve therapeutic levels, which can increase the risk of adverse effects [38,39,40]. Therefore, pharmaceutical scientists dedicate considerable effort to understand [41,42], predict [43,44], and manipulate [45,46] the solubility of drug molecules. Techniques such as salt formation, cosolvent systems, cyclodextrin-based formulations, and nanoparticle delivery systems are employed to enhance solubility and thereby improve drug performance [47,48,49,50]. As it was said, solubility modeling and predictions play a pivotal role in the pharmaceutical industry by aiding in drug discovery and development processes [28,51,52,53]. Accurate predictions of solubility help researchers in selecting the most promising drug candidates, optimizing formulation strategies, and improving drug delivery systems. Among various predictive methods, artificial neural networks, with their ability to learn complex patterns from data, have emerged as powerful tools in solubility modeling and found widespread use in the pharmaceutical domain [54,55,56,57]. The integration of advanced computational methods like neural networks with traditional pharmaceutical research facilitates the design of more effective and safer medications.

The abovementioned need for safer pharmaceutical products, not only in terms of patient safety but also from an environmental perspective, led to a widespread application of the “green chemistry” framework [58,59]. In particular, the introduction of green solvents in the pharmaceutical industry could be regarded as a paradigm shift towards sustainability and environmental responsibility [60,61]. Green solvents, characterized by their low toxicity, renewable sourcing, and biodegradability, offer a compelling alternative to traditional organic solvents, which often pose significant environmental and health hazards [62,63]. Among the various techniques and chemicals used to meet the criteria of the concept of green chemistry, a notable group are deep eutectic solvents, abbreviated as DESs [64,65,66,67,68]. The majority of these systems are mixtures of two or more components, which include a hydrogen bond acceptor (HBA) and a hydrogen bond donor (HBD) that can be either solid or liquid. The melting point of the resulting mixture is lower than the melting points of its components, because of which they remain in liquid form even at room temperature [69,70]. DESs exhibit a unique combination of properties such as low toxicity, biodegradability, and importantly tunable physicochemical characteristics [71,72,73]. Despite the fact that the extent of their environmental friendliness has recently become a topic of debate [74,75,76,77], DESs showcase remarkable potential across various domains of pharmaceutical applications, including drug synthesis, formulation, and extraction processes [78,79,80,81,82,83,84,85,86,87,88]. Noteworthily, DESs have been shown to be effective solubilizers of ibuprofen [89,90] and ketoprofen [91]. As it can be seen, a substantial dataset has emerged, which serves as an intriguing starting point for modeling the solubilization properties of both examined active substances. Nevertheless, it is worth enriching this collection with new experimental results, especially the solubility values in DESs.

The aim of this paper is threefold. Firstly, the tuning of ibuprofen and ketoprofen solubility by designed DESs was undertaken. Then, machine learning was used for the formulation of the solubility model. Finally, predictions of selected analogues in DESs comprising choline chloride and betaine were provided.

## 2. Results and Discussion

The experimental part of this project involved the optimization of DES content and compositions at room temperature to find the most suitable solvent for ibuprofen and ketoprofen. Then, the most effective solvent systems were systematically studied at different temperatures and by dilution with water. This aspect of adding water to the DES often leads to a surprising structure [92,93] and solubilizing properties [79,83]. Water is considered an antisolvent because the aqueous solubility of various compounds is several orders of magnitude lower compared to DESs, and dilution with water in excess amounts significantly reduces the solubility of many solutes in such DES–water mixtures. However, there are narrow ranges of water content that promote solubility compared to pure DES. Nonetheless, due to the complicated and complex structure of aqueous DES mixtures, water cannot be considered a cosolvent in such ranges. Using the example of deep eutectic choline chloride/urea/water solvent mixtures [92], it was shown that the DES nanostructure is formulated and preserved in a wide hydration range up to a remarkably high water content. This is attributed to the solvophobic sequestration of water in nanostructured domains around cholinium cations. At higher water concentrations, such separation becomes unfavorable, and the DES nanostructure is disrupted. That is why DESs are often highly hygroscopic, and the control of the water content is so important. If excess amounts of water are added, the water–water and DES–water interactions become dominant, and only in such a regime can DES–water mixtures be considered as aqueous solutions of DES components. The presence of these nanostructures might be regarded as the structural origin of the observed increased solubility of the dissolved substances compared to dry DES.

### 2.1. Solubility of IP and KP in DESs

In the initial phase of the experiments, a total of 36 eutectic systems were studied for both ibuprofen (IP) and ketoprofen (KP). One arrives at this number by combining two hydrogen bond acceptors (HBAs), namely, choline chloride (ChCl) and betaine (BI), with six hydrogen bond donors (HBDs), namely ethylene glycol (ETG), diethylene glycol (DEG), triethylene glycol (TEG), 1,3-butanediol (B3D), 1,2-propanediol (P2D), and glycerol (GLY), as well as three molar HBA:HBD proportions, namely 1:1, 1:2, and 1:4. This phase was aimed not only at determining the solubility of IP and KP in neat DES but also at selecting the best systems for next-stage studies involving aqueous mixtures. The composition screening was conducted at 25 °C.

In the case of ibuprofen, the following observations could be made. First of all, the eutectic systems involving choline chloride were slightly more effective than those utilizing betaine. Secondly, when comparing the effectiveness of HBD components, TEG turned out to be the most effective in almost all cases, the only exception being the ChCl-GLY among the 1:2 systems. Finally, when comparing molar ratios, the 1:2 ratio was the most efficient, with the 1:4 ratio coming second and followed by the 1:1 ratio. For both choline chloride and betaine in the 1:2 molar ratio, the following decreasing order of solubilizing potential was noticed: TEG > GLY > DEG > ETG > B3D > P2D. This order varies only slightly in other proportions, with GLY being the most effective in the case of 1:1 molar ratio, ETG surpassing DEG in the 1:4 molar proportion, and P2D outperforming B3D on two occasions. The actual solubility of IP at 25 °C in the ChCl-TEG (1:2) system was found to be x_IP_ = 0.353, while the ChCl-GLY (1:2) system amounted to a solubility of x_IP_ = 0.349. In the case of betaine, these values were x_IP_ = 0.321 for BI-TEG (1:2) and x_IP_ = 0.297 for BI-GLY (1:2). Detailed solubility values of IP in the studied systems can be found in the Supplementary Materials.

A quite similar picture arises when inspecting the results obtained for ketoprofen. Again, the eutectics formed with choline chloride achieved a higher solubility of KP than the ones with betaine. As in the case of IP, the 1:2 molar ratio was found to be the most efficient, followed by the 1:4 and 1:1 molar ratios, both in the case of choline chloride and betaine. There was, however, a difference in the effectiveness of the HBD compounds. In the case of choline chloride, TEG was the best-performing hydrogen bond donor in all molar ratios. For betaine, this position was taken by GLY, again in all molar ratios. The decreasing solubility order of KP in eutectics with choline chloride is the following: TEG > DEG > GLY > ETG > B3D > P2D, with glycerol replacing DEG in the 1:4 molar ratio. On the other hand, for the systems involving betaine, the order is GLY > TEG > DEG > ETG > B3D > P2D, with ETG replacing DEG in the 1:4 molar ratio. The solubilities of KP among the most effective systems for both choline chloride and betaine were x_KP_ = 0.067 for ChCl-TEG (1:2), x_KP_ = 0.058 for ChCl-DEG (1:2), x_KP_ = 0.062 for BI-TEG (1:2), and x_KP_ = 0.060 for BI-TEG (1:2). Detailed KP solubility values for the considered systems are presented in the Supplementary Materials.

Based on the performed initial study, the two best-performing eutectic systems were selected for further solubility measurements, one involving choline chloride (ChCl-TEG 1:2 for both IP and KP) and one with betaine (ChCl-TEG 1:2 for IP and ChCl-GLY 1:2 for KP). These systems were used in conjunction with water, forming ternary aqueous mixtures with varying amounts of DESs. The studies involved several temperatures ranging from 25 °C to 40 °C, and it was no surprise that elevated temperatures promoted the dissolution of the studied compounds. Again, it should be noted that the complex nature of aqueous DES systems means that they cannot be described in terms of simple cosolvency or antisolvency effects. Nonetheless, these apparent effects can still be regarded as a useful way of presenting and describing solubility data.

**Figure 1:**
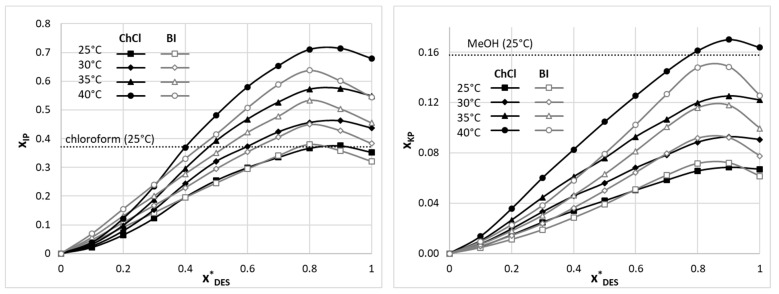
The solubility curves of ibuprofen (IP, **left** panel) and ketoprofen (KP, **right** panel) in aqueous DES mixtures at various temperatures, expressed as solvent-composition-related mole fractions. ChCl stands for eutectics with choline chloride, and BI stands for eutectics with betaine. x*_DES_ stands for mole fractions of solute-free DES in aqueous mixtures. For comparison, the room-temperature solubilities of IP in chloroform [94,95] and KP in methanol [96] are provided as reported in the literature.

For both IP and KP, regardless of the applied DES, the addition of the eutectic to water increases the solubility of the active pharmaceutical ingredient up to a certain point at which the solubility is greater than in the neat DES. After achieving this infliction point, the solubility decreases to the level of the neat DES. Hence, it is useful to describe this behavior as an apparent cosolvency/antisolvency effect. The details of the solubility curves, however, vary among the studied systems and are presented in Figure 1. For both IP and KP, the systems involving choline chloride are characterized by the highest solubility obtained in the x*_DES_ = 0.9 composition. At 25 °C, the solubility of IP in the ChCl-TEG (1:2, x*_DES_ = 0.9) system was found to be x_IP_ = 0.375, while the solubility of KP in the analogous system was x_KP_ = 0.068. On the other hand, when inspecting the solubility of ibuprofen in betaine-containing aqueous DES mixtures, it was found that the optimal composition is x*_DES_ = 0.8. At this point, the IP solubility in the BI-TEG (1:2, x*_DES_ = 0.8) system is x_IP_ = 0.380, which, interestingly, is greater than for the optimal system involving choline chloride. In the case of ketoprofen solubility, using aqueous mixtures of DESs with betaine results in the optimal composition of x*_DES_ = 0.9, although the difference in solubility with the x*_DES_ = 0.8 composition is less than 1%. For the BI-GLY (1:2, x*_DES_ = 0.9) system, the KP solubility equals x_KP_ = 0.072 at 25 °C. Detailed solubility values can be obtained from the Supplementary Materials.

**Figure 2:**
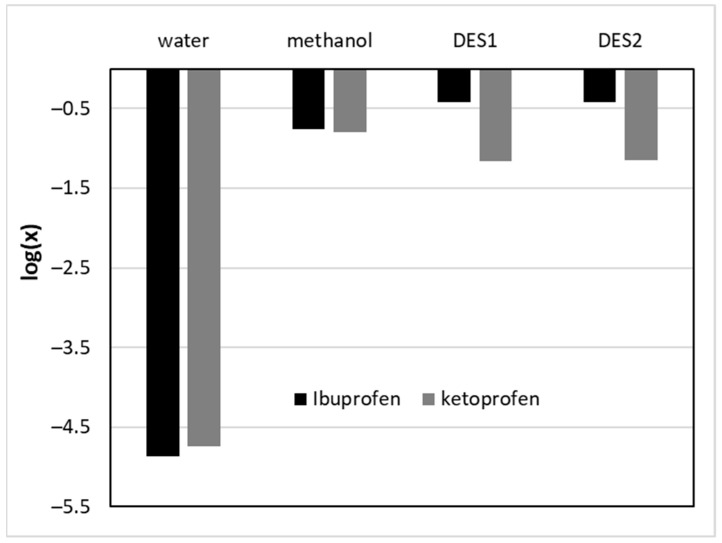
Comparison of IP and KP solubility in the most efficient DES and selected non-DES solvents at room temperature. DES1 and DES2 denote aqueous DES mixtures. For ibuprofen, DES1—ChCl + TEG 1:2 (x*_DES_ = 0.9); DES2—BI + TEG 1:2 (x*_DES_ = 0.8). For ketoprofen, DES1—ChCl + TEG 1:2 (x*_DES_ = 0.9); DES2—BI + TEG 1:2 (x*_DES_ = 0.9).

When comparing the obtained results with the literature data, it is evident that in the case of ibuprofen, the optimal aqueous DES mixtures outperform even the most efficient organic solvent, namely, chloroform, characterized by an IP solubility equal to x_IP_ = 0.371 at 25 °C [94,95]. The difference is even greater when comparing the solubilities achieved in other most efficient organic solvents, such as octanol, dichloromethane, or acetone. Furthermore, chloroform can hardly be considered a green solvent, which makes the usage of the studied DES systems even more attractive. On the other hand, such high solubilization efficiency was not achieved in the case of ketoprofen. In this case, the optimal DES–water composition was outperformed even by methanol, which resulted in the solubility of KP equal to x_IP_ = 0.158 at 25 °C [96]. The difference in the DESs’ performance is visualized in Figure 2, which shows the logarithmic mole fraction solubility of the two studied compounds in various solvents. In general, ketoprofen is characterized by a lower solubility than ibuprofen in all systems, although in the case of water, the solubilities are comparably low. Applying DESs as solubilization media drastically increases the solubility compared to water in both cases. The differences between the two compounds are evident, however, when looking at the efficiency of DESs compared to methanol. While for ibuprofen they offer a substantial solubility advantage, their performance in the case of ketoprofen is not optimal.

### 2.2. Solubility Dataset

**Figure 3:**
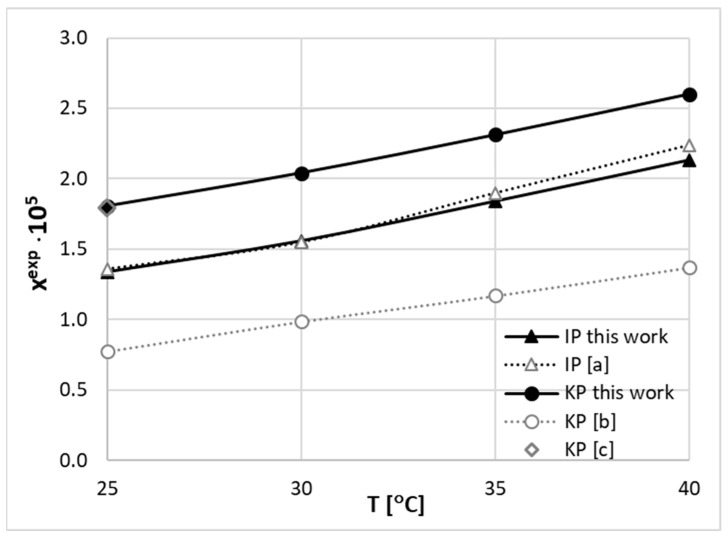
The temperature dependence of IP and KP solubility in water. The newly measured data presented in this work were augmented with already published results, namely, [a] by Manrique et al. [104], [b] by Gantiva et al. [107,108], and [c] by Jiménez et al. [105].

The experimentally determined solubility of both studied COX inhibitors proved the effectiveness of the DES design for optimizing the solubilizers’ performance. It is interesting to provide a broader overview of the distribution of this physical property based on already published data. Hence, a careful literature search was undertaken to extend the solubility dataset. This was conducted by the inclusion of reported solubility in neat solvents and binary solvent mixtures. In the case of IP, such data are available for 18 neat solvents, including methanol [94], ethanol [94,97,98,99,100], 1-propanol [97], 2-Propanol [94,97,101,102], 1-butanol [97], isobutanol [97], 1-pentanol [97], 3-methyl-1-butanol [97], 1-octanol [95,100], propylene glycol [98], ethyl acetate [94,97], isopropyl acetate [51], isopropyl myristate [51], acetone [94,103], 2-butanone [51], 4-methyl-2-pentanone [94], 3-methyl-2-butanone [51], acetonitrile [51], cyclohexane [95], toluene [94], heptane [51], dichloromethane [103], chloroform [94,95], and water [99,104]. Additionally, there are four reports documenting the solubility of IP in binary mixtures such as water–methanol [105], water–ethanol [99], water–propylene glycol [104], and ethanol–propylene glycol [98]. The solubility of S-enantiomer, (S)-(+)-ibuprofen, was measured [106] in methanol, 1-butylanol, isobutanol, 1-pentanol, and 1-octanol. The saturated solutions of ketoprofen were much less intensively studied, and there is only one comprehensive study [96] providing the solubility in the following neat solvents: methanol, isopropanol, n-butanol, acetonitrile, ethyl acetate, 1,4-dioxane, and toluene. The work by Soto et al. [96] is particularly interesting since the authors compared three different methods of experimental solubility determination. The concluded consensus between measured solubility data makes this report highly reliable. There are also other reports by Gantiva et al. [107,108] providing the solubility of KP in aqueous binary mixtures of ethanol and propylene glycol. However, the reliability of the provided values is debatable. Indeed, the reserve of these data was already emphasized by Filippa et al. [109]. Serious discrepancies were noticed and highlighted, suggesting that the aqueous solubility of ketoprofen at 298.15 K [107,108] (4.295 × 10^−4^ mol/L) is much lower than the value published by Yalkowsky et al. [110], which is 5.58 × 10^−3^ mol/L. The same reserve applies to the solubility of ketoprofen in cyclohexane, since, as reported in [111], the solubility of ketoprofen in cyclohexane is 5.50 × 10^−4^ mol/L at 298 K, while according to the study of Filippa et al. [109], the solubility of this compound is 4.16 × 10^−3^ mol/L at 300.15 K. Also, the solubility value determined for propylene glycol seems problematic, since according to the measurements of Filippa et al. [109], the solubility of ketoprofen in this solvent is 0.499 mol/L (300.15 K), while the value reported by Gantiva et al. [107,108], for similar temperature conditions (298.15 K), was 0.1832 mol/L. In order to resolve the discrepancies and provide a consistent set of solubility in the DES–water systems studied here, new measurements of both IP and KP in water were performed. The obtained data are shown in Figure 3, where the results of other studies are also provided.

**Figure 4:**
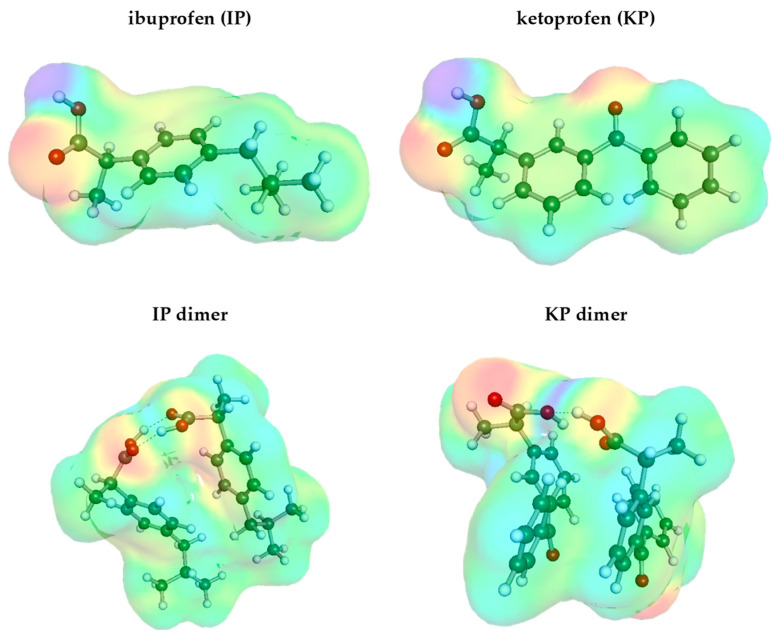
The distributions of electron charge density of IP (**left**) and KP (**right**) molecules, as well as their dimers resulting from self-association.

As evidenced by the plots in Figure 3, there is a pretty solid consensus on IP solubility in water. The deviations between our measurements and those by Manrique et al. [104] are marginal and within standard deviations. To the contrary, serious discrepancies are observed for the solubility of KP in water. It happened that the results of our measurements agree with data provided by Jiménez et al. [105] for ambient conditions and are seriously higher compared to data provided by Gantiva et al. [107,108]. This raises the question whether the latter values correspond to the equilibrium state of the KP–water system. Although the congruency of solubility at 25 °C between our measurements and the available literature might serve as an argument, one can obtain additional clues based on the electron charge distributions of IP and KP molecules. As provided in Figure 4, the electron density distributions for both studied compounds are characterized mostly by non-polar regions (green), with the exception of the carboxylic group, which induces local positively (blue) and negatively (red) charged surfaces due to the presence of hydroxyl and keto groups, respectively. KP also has an additional negatively charged region due to the presence of the oxygen atom in the moiety. Hence, the observed low solubility of both COX inhibitors in water is due to the mostly apolar nature of these compounds. However, it seems to be rational to expect a higher solubility of KP in water compared to IP, as the oxygen atom can be actively involved in hydration. In addition, it is worth adding that both compounds are able to form stable dimers, which is rather expected bearing in mind that they belong to the group of carboxylic acids. Hence, as a result, self-association is highly probable, and stabilization is to be granted from the cyclic structure of the $C_{2}^{8}$ type, meaning that the two hydrogen bonds are involved in an eight-center ring creation. The occurrence of such strong associations is typical for all carboxylic acids. The involvement of carboxylic groups of two IP molecules is particularly important, since these are the only polar centers that might be involved in direct contact with polar solvent molecules. Indeed, as it is documented in Figure 4, a strong hydrogen bond is formed between the donating hydroxyl group of IP and the accepting oxygen atom in water. Since the rest of the molecule is non-polar, it is expected that the polarity of the IP dimer will be much smaller compared to the monomer. A similar pattern can also be noticed for ketoprofen. In either case, the dominant surface exposed for contacts with solvent molecules is mostly non-polar, and the involvement of a carboxylic group in the dimer formation further reduces the net polar region available for contacts with polar molecules.

Also, in the case of dimers, ketoprofen seems to be more prone to interactions with polar solvents, since the oxygen atom attached to the carbon atom chain is not affected by dimerization. Hence, both qualitative analysis of electron charge distributions and congruency between our measurements and the ones provided by Jiménez et al. [105] suggest that the reports by Gantiva et al. [107,108] should be taken with reserve.

This conclusion drawn from the above discussion is important from the perspective of machine learning, since the quality of the trained model is crucially dependent on the consistency and representativeness of the data used for training purposes. That is why the questioned data were not included in the training dataset. However, to make the model as reliable and as general as possible, the dataset was extended by the inclusion of compounds belonging to the same group. This enabled the capture of a wider range of correlations between measurements and molecular features. There is, however, a limitation to the choice, since two criteria must be met, namely, not only reliable solubility data should be included, but fusion data must also be available. The latter requirement is related to the fact that the molecular descriptor of the first choice is the value of solubility computed using COSMOtherm (version 24.0.0). This seriously restricts the potential scope of compounds to be included, and, to our best knowledge, the solubility and fusion data were only recorded for two closely related compounds. The first one, flurbiprofen, was studied in 1-octanol [100,112], ethanol [100], and hexane [112]. For the second one, dexibuprofen, there are available solubility values reported by Wang et al. [106] for methanol, n-butyl alcohol, isobutyl alcohol, n-amyl alcohol, and n-octyl alcohol. Hence, these data were included in the solubility training dataset.

### 2.3. Machine Learning Model

The solubility model was constructed based on the best-performing regressors identified after comprehensive hyperparameter tuning and validation. During model selection, a multi-layer perceptron (MLP) regressor achieved significantly higher accuracy than other nonlinear algorithms for the COX inhibitors dataset. Consequently, the ensemble formulation step simply selected the best individual model, which was the MLP regressor.

The MLP model architecture was optimized in a flexible manner by allowing for the adjustment of both parameter values and the overall network structure. The optimal network consisted of an input layer, multiple hidden layers of variable sizes, and an output layer. Hyperparameter optimization was performed extensively using the Optuna Study framework. The best-performing model had the following hyperparameters: hidden layer sizes of 64, 17, 45, 54, 18, 14, 45, and 61, rectified linear unit (ReLU) activation, L2 regularization (alpha) of 0.045, an adaptive learning rate, a limited-memory Broyden–Fletcher–Goldfarb–Shanno (L-BFGS) solver, and 928,539 maximum iterations.

The multiple hidden layers enable complex nonlinear mapping between inputs and outputs. Larger initial layers (64–45 neurons) capture richer feature representations from descriptors, while smaller later layers (14–45 neurons) perform higher-level abstraction. The ReLU activation introduces nonlinearity while avoiding vanishing gradients. Adaptive learning optimizes the learning rate during training. An L2 regularization of 0.024 prevents overfitting by penalizing model complexity. The optimized MLP architecture and hyperparameters performed best for learning the solubility structure–property relationships from molecular fingerprints in a nonlinear fashion. As the most efficient optimizer, L-BFGS was selected as it can reach a local minimum gradient-based quasi-Newton style. The high maximum iteration count ensured convergence.

**Figure 5:**
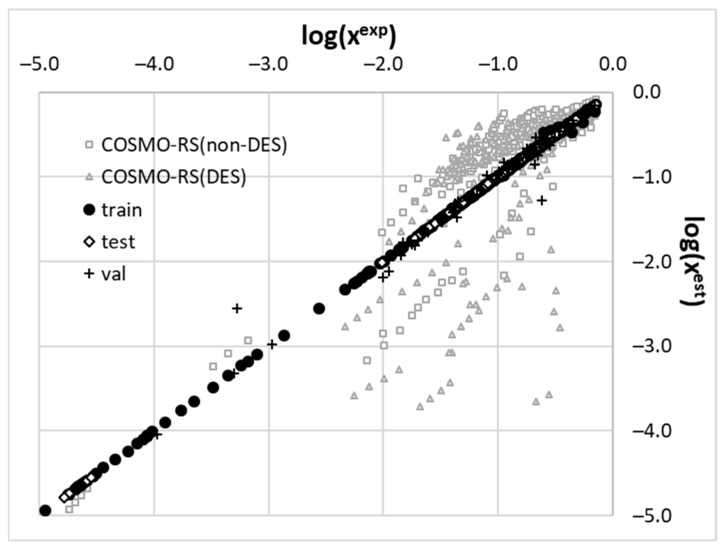
The correlation between experimental and computed solubility values of COX inhibitors in DES and non-DES systems using the developed MLP model and COSMO-RS approach. The accuracy of the MLP model is as follows: RMSD(train) = 0.016, RMSD(test) = 0.118, and RMSD(validation) = 0.136.

The accuracy of the formulated model is graphically illustrated in Figure 5, which documents the correlation between experimental and computed solubility expressed as decadal logarithms. The three dataset splits, namely, training (N = 406), test (N = 88), and validation (N = 87), are plotted in black ink to distinguish the solubility value distributions determined by COSMO-RS for non-DES and DES systems. It is clearly visible that the back-computed values perfectly match the experimental data. This is not the case for solubility derived directly from COSMO-RS. Neither of the two divergent groups, non-DES and DES systems, can be characterized accurately enough by this approach alone. In the case of solubility, in many DESs, the analyzed solutes are predicted with deviations of several orders of magnitude in mole fraction. However, despite such inaccuracies, the utilization of COSMO-RS-computed solubility remains a valuable source of information for the nonlinear models. This directly justifies the adopted methodology herein. It is worth noting that excluding the computed solubility values from the pool of molecular descriptors led to models with much lower accuracy than the one presented in Figure 5.

**Figure 6:**
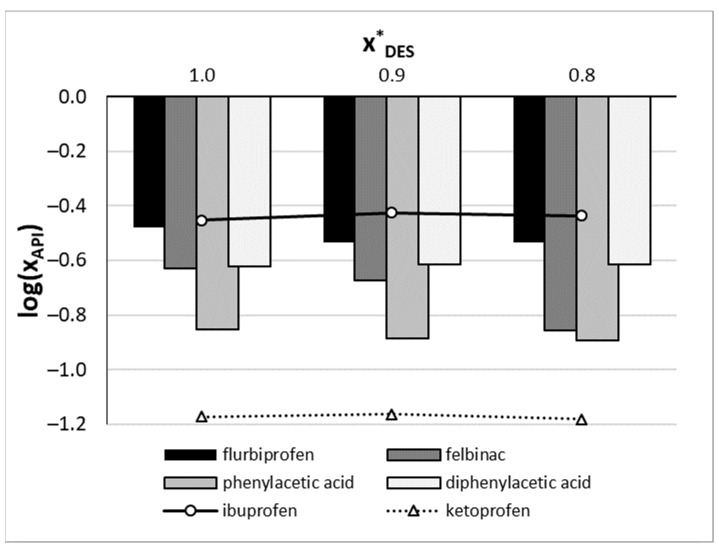
Results of predictions made by the solubility model for new closely related compounds at room temperature. DES stands for the deep eutectic involving choline chloride and TEG in a 1:2 molar ratio. Different compositions of the aqueous mixture are presented.

Based on the obtained model, a new prediction was made for selected analogues of IP and KP. As mentioned in the methodology section, this step is limited to those compounds for which solubility calculations are possible, i.e., for which fusion data are available. Four closely related compounds were therefore selected, namely, flurbiprofen, felbinac, phenylacetic acid, and diphenylacetic acid. For these compounds, the values of the molecular descriptors were calculated in the same way as for the training dataset, and the prediction was made using optimized values of the MLP architecture. As a result, the hypothetical solubilities were obtained, as shown in Figure 6. Based on these predictions, it is expected to be beneficial to perform actual solubility measurements for all screened analogues, as the solubility is likely to be improved in the choline chloride–TEG deep eutectic mixture. The highest solubility is expected for flurbiprofen in this DES.

## 3. Materials and Methods

### 3.1. Materials

Two chemical compounds, namely, racemic ibuprofen (IBU, CAS: 15687-27-1, MW = 206.28 g/mol) and racemic ketoprofen (KET, CAS: 22071-15-4, MW = 254.28 g/mol), were used as the main objects of this study. Both were supplied by Sigma Aldrich (Saint Louis, MO, USA) and had a purity of ≥98%. As the constituents of deep eutectic solvents, the following compounds were used: choline chloride (ChCl, CAS: 67-48-1), betaine (BI, CAS: 107-43-7), ethylene glycol (ETG, CAS: 107-21-1), diethylene glycol (DEG, CAS: 111-46-6), triethylene glycol (TEG, CAS: 112-27-6), glycerol (GLY, CAS: 56-81-5), 1,2-propanediol (P2D, CAS: 57-55-6), and 1,3-butanediol (B3D, CAS: 107-88-0). All the above chemicals were similarly obtained from Sigma Aldrich (St. Louis, MO, USA) and had a >99% purity. Also, methanol (CAS: 67-56-1) with a ≥99% purity supplied by Avantor Performance Materials (Gliwice, Poland) was used as a secondary solvent. Apart from choline chloride, which was dried before use, all other compounds were used as supplied without any additional procedures.

### 3.2. Preparation of the Calibration Curves

Stock solutions of ibuprofen and ketoprofen were prepared in order to prepare the two calibration curves in 100 mL volumetric flasks using methanol as a solvent. Subsequent dilutions of these initial solutions were made by transferring fixed amounts of the stock solution into 10 mL volumetric flasks and adding appropriate amounts of methanol. In this way, two series of solutions with decreasing concentrations were obtained. A total of eleven solutions were used, and their concentrations ranged from 0.0126 mg/mL to 0.0504 mg/mL in the case of ibuprofen and from 0.01008 mg/mL to 0.02016 mg/mL in the case of ketoprofen. These solutions were measured using an A360 spectrophotometer from AOE Instruments (Shanghai, China) in the wavelength range from 200 nm to 500 nm. The characteristic wavelengths corresponding to the absorbance maxima were found to be 223 nm and 254 nm for ibuprofen and ketoprofen, respectively. For each of the compounds, separate curves were prepared and averaged. The linear regression equations were obtained as A = 27.268 × C + 0.001 for ibuprofen and as A = 68.792 × C + 0.002 for ketoprofen. In order to validate the curves, the R^2^ determination coefficients, limits of detection (LODs), and limits of quantification (LOQs) were calculated. For the ibuprofen curve, the following parameters were obtained: R^2^ = 0.9968, LOD = 0.00233 mg/mL, and LOQ = 0.00698 mg/mL. For the ketoprofen curve, these parameters were R^2^ = 0.9979, LOD = 0.00052 mg/mL, and LOQ = 0.00156 mg/mL. The above values indicate the adequacy of the prepared curves for measuring the solubility of the two considered compounds.

### 3.3. Sample Preparation and Solubility Measurements

For the determination of the solubilities of ibuprofen and ketoprofen in the studied systems, the well-known and widely used shake-flask method was applied. This method has been used numerous times by many researchers, including our research team [113,114,115,116], and has proven its reliability through the comparison of obtained solubility values with the literature data. The choice of the eutectic components was based on our previous experiences, which resulted in the elevated solubility of many APIs [79,83,115].

The formulation of deep eutectic solvents involved using either choline chloride or betaine as a hydrogen bond acceptor (HBA) and one of six studied polyols, namely, TEG, DED, ETG, GLY, P2D, or B3D, as a hydrogen bond donor (HBD). The preparation of a particular DES system involved mixing the appropriate HBA with a selected HBD in glass vessels in appropriate molar ratios. Such mixtures were then placed on a heating plate and further mixed until they formed a homogenous solution. The created DESs were used both in their neat form and in aqueous mixtures with specifically calculated amounts of both water and eutectic.

The first step in the determination of the solubilities of ibuprofen and ketoprofen in the studied systems was the preparation of their saturated solutions. In order to achieve this, an excess amount of the compound was placed in a test tube, which was later filled with the pure DES or water–DES mixture. The solubilities were measured at 25 °C, 30 °C, 35 °C, and 40 °C. In order to achieve a stable temperature, the Orbital Shaker Incubator ES-20/60 from Biosan (Riga, Latvia) was used. The samples were incubated for 24 h with simultaneous mixing at 60 rev/min. In the next step, the samples were filtered using a syringe equipped with a 0.22 µm pore size PTFE filter. To prevent precipitation, the test tubes, syringes, pipette tips, and filters were initially heated at the same temperature as the measured sample. In order to remain in the linearity range of the curve, the filtered samples were diluted with methanol before spectrophotometric measurements. The spectra of the samples were recorded on the A360 spectrophotometer, with a 200 nm–500 nm wavelength range and a 1 nm resolution. Methanol was used for the calibration of the spectrophotometer. The absorbance at characteristic wavelengths, i.e., 223 nm for ibuprofen and 254 nm for ketoprofen, was recorded, and the concentration of the compounds in the samples was calculated based on the calibration curve obtained earlier. For the determination of the mole fractions of both compounds, the density of the samples was additionally determined. The procedure relied on weighing 1 mL of each solution in a 10 mL volumetric flask. For this task, a RADWAG (Radom, Poland) AS 110 R2.PLUS analytical balance was used with 0.1 mg precision. Three samples were prepared and measured for each system, and the final values are a result of averaging. In order to evaluate the quality of the obtained results, the standard deviation values were calculated. All experimental results are provided in Supplementary Tables S1 and S2.

### 3.4. Machine Learning Protocol

The training of nonlinear models was performed according to the scheme already applied to our previous projects [79,83,117,118]. Since details were already presented, here, only a brief synopsis is provided. Based on Python code, the comprehensive series of regressors were trained by means of hyperparameter tuning. The solubility prediction model was crafted utilizing in-house-developed Python (version 3.10, https://www.python.org/) code tailored for hyperparameter tuning across 36 regression models. These models encompass diverse algorithms, spanning linear models, boosting, ensembles, nearest neighbors, neural networks, and other regressor types. The hyperparameter space was meticulously explored to identify optimal values using the Optuna study (version 3.2, https://optuna.org/), an open-source Python package for hyperparameter optimization [119]. Model refinement involved 5000 minimization trials employing the tree-structured Parzen estimator (TPE) as the search algorithm sampler. To assess each regression model’s performance, a custom score function was defined, amalgamating multiple metrics to gauge accuracy and generalizability, as elucidated in a prior work [118]. The scoring function notably incorporates penalties derived from learning curve analysis (LCA) performed using the scikit-learn 1.2.2 library during parameter tuning. Due to the computational intensity of LCA, only two-point computations were executed, encompassing 50% and 100% of the total data. Subsequently, LCA evaluations of the final model entailed 20-point calculations within the 50–100% data range. The values integrated into the custom loss function correspond to the mean MAE values obtained at the largest training set size. Thus, the custom loss function integrates both accuracy and generalizability aspects, providing insights into the model’s performance on unseen data. After determining the optimal parameters for each model, the highest-performing ones were selected for ensemble construction, wherein the weights of individual model contributions to the final solubility prediction were optimized. These weight values were derived through the minimization of the root mean square deviation between the predictions for the validation subset and the observed values. The efficacy of the final ensemble model was subsequently assessed using the test subset to ascertain its accuracy. All models which contribute less than 1% are not included in the ensemble.

### 3.5. Molecular Descriptors

The molecular information encoded in the appropriate series of descriptors is crucial from the perspective of an adequate representation of the relationship between measurements and features used for modeling. There are a variety of approaches intended to probe the molecular hyperspace from different perspectives. This project follows the main tenet that temperature- and concentration-dependent molecular characteristics are to be used for solubility modeling. This initial bias rules out many approaches for molecular descriptor determination, including SMILES-related methods, fingerprints, and also the direct utilization of many first-principle procedures. However, there is a very promising and well-recognized methodology combining both quantum chemistry computations and statistical mechanics to account for environmental influences on the thermodynamic properties of the systems under consideration. This approach, acronymed as COSMO-RS (COnductor-like Screening Model for Realistic Solvents) [120], is designed for bulk systems, in principle, of arbitrary compositions, concentrations, and temperature ranges. That is why this particular framework is selected as a default source of theoretically determined molecular characteristics, as it was already successfully documented in our previous projects [79,83,117,118,121,122,123,124]. In particular, the COSMO-RS theory implemented in the COSMOtherm package (version 24.0.0) offers a very convenient way to saturate system characteristics and is used as the source of molecular descriptors also in this project.

The first choice of molecular descriptors is the solubility computed with the aid of COSMOtherm. Unfortunately, this imposes limitations inherent to the approach, and its utilization requires providing the fusion data, such as heat of fusion, temperature of melting, and eventually the heat capacity change upon melting. These experimental parameters are crucial for including the lattice energy. Fortunately, these data are available for both solutes, and several estimates were reported of the melting temperature and heat of fusion. The following mean values were adopted in this work, based on the compilation of data from various sources, as available in the literature [125]: T_m_ = 348.64 K and ΔH_m_ = 26.69 kJ/mol for ibuprofen and dexibuprofen, T_m_ = 367.99 K and ΔH_m_ = 25.52 kJ/mol for ketoprofen, and T_m_ = 387.34 K and ΔH_m_ = 27.86 kJ/mol for flurbiprofen. The COSMOtherm program [126] was used for computing solubility using the newest BP_TZVPD_FINE_24.ctd parametrization. All structures of both solutes and solvent molecules were characterized by conformational diversity derived by comprehensive conformational analysis using the COSMOconf program (version 24.0.0) [127]. Solubilities were computed by fully solving the Solid–Liquid Equilibria (SLE) problem rather than using an iterative procedure, since it fails for highly soluble cases.

**Figure 7:**
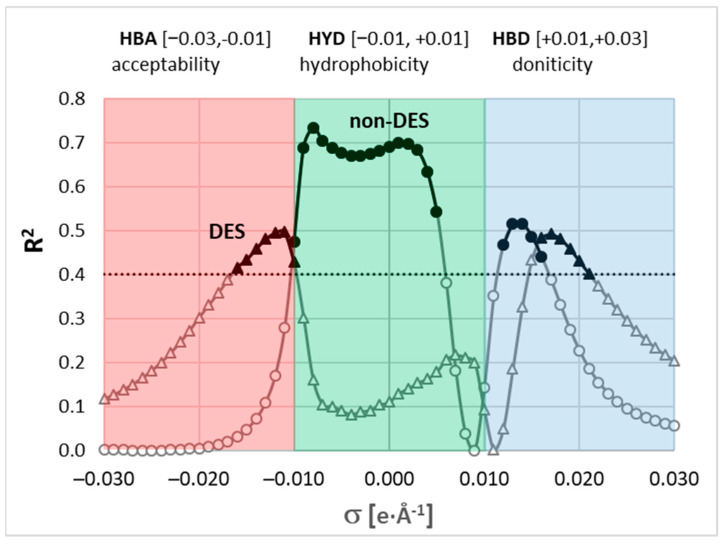
Correlation between relative solute–solvent σ-potentials and experimental solubility, expressed as a function of σ values. Two series represent correlations computed for subsets including only non-DES solvents (neat solvents and binary mixtures) or DES-only systems. Bold symbols define regions used as a source of molecular descriptors, where R^2^ > 0.4 for either subset.

The second type of molecular information was taken from σ-potential. These values were selected rather than σ-profiles since they are temperature-related. For each solute and solvent, the σ-potential profiles were computed for a pure single-component state at given temperature. The set of molecular descriptors used for machine learning was computed as the difference between the σ-potential of a pure solute and the σ-potential of a solvent at a given temperature. In the case of multicomponent solvents, the σ-potential was represented as the value weighted by the mole fraction of the solute-free mole fraction and then used for relative σ-potential profile determination. Typically, the COSMOtherm generates σ-potential profiles in the form of 61 points for σ between −0.03 and +0.03 e/Å^2^ with 0.001 step. To reduce the number of descriptors to the most promising, the relationship between the experimental solubility and values of relative σ-potentials was inspected and quantified by R^2^ values for every σ. This was conducted for two subsets, namely, only non-DES solvents, including neat ones and binary mixtures, and DES-only systems. The obtained results are provided in Figure 7. It documents that there is a modest correlation between experimental solubility and relative σ-potential profiles. However, the regions with the highest contribution are different for both subsets. For non-DES systems, the most significant is the region of σ ∊ [−0.01, +0.01] typically attributed to hydrophobicity (HYD) and, to a lesser extent, the fraction of the hydrogen bond donicity (HBD) defined by σ ∊ [+0.01, +0.03]. From the solubility perspective, the hydrogen acceptability (HBA) σ ∊ [−0.03, −0.01] is marginal in the case of non-DESs. Conversely, for DES systems, both HBD and HBA regions have the highest correlation with experimental solubility, and the contribution of the HYD region is marginal. This suggests that the total solubility dataset is very diverse and covers quite a large molecular structure representation. To include the diversity into the training protocol and, at the same time, reduce the number of data points of the relative σ–potentials, only such values were used which fulfilled the criterion of R^2^ > 0.4 for either subset. This is marked by bold points in Figure 7. Hence, the values of computed σ-potentials within the range of σ between −0.017 and +0.021 with the exclusion of the region between 0.006 and 0.011 were used as molecular descriptors.

## 4. Conclusions

This project experimentally and theoretically screened solvent systems for enhancing the solubility of two COX inhibitors: ibuprofen and ketoprofen. Experimental DES formulations were optimized by varying solvent composition and content. Thirty-six DESs were explored by combining two hydrogen bond acceptors (choline chloride, betaine) with six donors (ethylene glycol, diethylene glycol, triethylene glycol, 1,3-butanediol, 1,2-propanediol, glycerol) at molar ratios of 1:1, 1:2, and 1:4. The most effective systems contained triethylene glycol at an HBA:HBD ratio of 1:2. Aqueous DES mixtures exhibited a pseudo-cosolvency effect, increasing solubility up to 0.2 mole fraction water content. This phenomenon is commonly observed due to water’s role in stabilizing solute-rich nanodomains around cholinium cations.

For ibuprofen, the optimal aqueous DES outperformed the most effective organic solvent (chloroform), offering a more sustainable alternative. In contrast, ketoprofen solubility in DES–water was surpassed by methanol. Further DES optimization is warranted.

A machine learning model using COSMO-RS descriptors accurately predicted the solubility for test compounds. This approach enables rational DES formulation design and solubility screening to advance pharmaceutical applications, circumventing laborious experimental screening.

## Data Availability

All data supporting the reported results are available on request from the corresponding author.

## Supplementary Materials

The following supporting information can be downloaded at https://www.mdpi.com/article/10.3390/molecules29102296/s1, Table S1: Mole fraction solubility of ibuprofen (IP) in neat DES containing either choline chloride or betaine as an HBA and one of six studied polyols as an HBD in various molar proportions at 25 °C. Standard deviation values are given in parentheses. Table S2: Mole fraction solubility of ketoprofen (KP) in neat DES containing either choline chloride or betaine as an HBA and one of six studied polyols as an HBD in various molar proportions at 25 °C. Standard deviation values are given in parentheses. Table S3: Mole fraction solubility of ibuprofen (IP) in aqueous DES mixtures at various temperatures. In the first column, x*DES denotes the mole fraction of DES in solute-free mixtures with water. Standard deviation values are given in parentheses. Table S4: Mole fraction solubility of ketoprofen (KP) in aqueous DES mixtures at various temperatures. In the first column, x*DES denotes the mole fraction of DES in solute-free mixtures with water. Standard deviation values are given in parentheses.
