# Supplementary material for: Experimental and Machine-Learning-Assisted Design of Pharmaceutically Acceptable Deep Eutectic Solvents for the Solubility Improvement of Non-Selective COX Inhibitors Ibuprofen and Ketoprofen
Source: Molecules. 2024 May 14;29(10):2296. doi: 10.3390/molecules29102296 (PMC11124057; doi:10.3390/molecules29102296)
Supplement: Supplementary file 1 [file molecules-29-02296-s001.zip › molecules-3009308-supplementary.pdf]

<u>Supplementary Materials</u>

Experimental and machine-learning-assisted design of pharmaceutically acceptable deep eutectic solvents for the solubility improvement of non-selective COX inhibitors ibuprofen and ketoprofen

Table of Content

Table S1. Mole fraction solubility of ibuprofen (IP) in neat DES containing either choline chloride or betaine as a HBA and one of six studied polyols as a HBD in various molar proportions at 25°C. Standard deviation values are given in parentheses. 2

Table S2. Mole fraction solubility of ketoprofen (KP) in neat DES containing either choline chloride or betaine as a HBA and one of six studied polyols as a HBD in various molar proportions at 25°C. Standard deviation values are given in parentheses. 2

Table S3. Mole fraction solubility of ibuprofen (IP) in aqueous DES mixtures at various temperatures. In the first column, x^*_{DES} denotes the mole fraction of DES in solute-free mixtures with water. Standard deviation values are given in parentheses. 3

Table S4. Mole fraction solubility of ketoprofen (KP) in aqueous DES mixtures at various temperatures. In the first column, x^*_{DES} denotes the mole fraction of DES in solute-free mixtures with water. Standard deviation values are given in parentheses. 4

Table S1. Mole fraction solubility of ibuprofen (IP) in neat DES containing either choline chloride or betaine as a HBA and one of six studied polyols as a HBD in various molar proportions at 25°C. Standard deviation values are given in parentheses.

	molar proportions and HBA		
	1:1	1:2	1:4
**HBD**	**choline chloride**		
P2D[a]	(0.1611±0.0024)	(0.2068±0.0046)	(0.1851±0.0016)
DEG[b]	(0.2420±0.0053)	(0.3149±0.0031)	(0.2902±0.0038)
TEG[c]	(0.2627±0.0052)	(0.3528±0.0029)	(0.3250±0.0046)
B3D[d]	(0.1584±0.0030)	(0.212±0.00270)	(0.1899±0.0025)
GLY[e]	(0.2793±0.0019)	(0.3495±0.0031)	(0.3187±0.0038)
ETG[f]	(0.2167±0.0034)	(0.3072±0.0025)	(0.2905±0.0061)
**HBD**	**betaine**		
P2D[a]	(0.1561±0.0027)	(0.1919±0.0023)	(0.2062±0.0014)
DEG[b]	(0.223±0.0026)	(0.2786±0.0026)	(0.2562±0.0039)
TEG[c]	(0.2589±0.0033)	(0.3209±0.0024)	(0.2973±0.0037)
B3D[d]	(0.1626±0.003)	(0.2001±0.0022)	(0.2035±0.0046)
GLY[e]	(0.2479±0.0025)	(0.2968±0.0037)	(0.2697±0.0091)
ETG[f]	(0.2182±0.0026)	(0.2758±0.0099)	(0.2576±0.0083)

a – 1,2-propanediol, b – diethylene glycol, c – triethylene glycol, d – 1,3-butanediol, e – glycerol, f – ethylene glycol

Table S2. Mole fraction solubility of ketoprofen (KP) in neat DES containing either choline chloride or betaine as a HBA and one of six studied polyols as a HBD in various molar proportions at 25°C. Standard deviation values are given in parentheses.

	molar proportions and HBA		
	1:1	1:2	1:4
**HBD**	**choline chloride**		
P2D[a]	(0.0337±0.0005)	(0.0428±0.0011)	(0.0364±0.0005)
DEG[b]	(0.0427±0.0006)	(0.0581±0.0015)	(0.0492±0.0006)
TEG[c]	(0.0453±0.0005)	(0.0670±0.0014)	(0.0564±0.0006)
B3D[d]	(0.0356±0.0005)	(0.0465±0.0009)	(0.0411±0.0008)
GLY[e]	(0.0400±0.0006)	(0.0577±0.0008)	(0.0494±0.0006)
ETG[f]	(0.0378±0.0005)	(0.0531±0.0007)	(0.0449±0.0005)
**HBD**	**betaine**		
P2D[a]	(0.0307±0.0003)	(0.0394±0.0004)	(0.0413±0.0007)
DEG[b]	(0.0395±0.0003)	(0.0516±0.0006)	(0.0490±0.0005)
TEG[c]	(0.0431±0.0006)	(0.0602±0.0007)	(0.0535±0.0009)
B3D[d]	(0.0334±0.0006)	(0.0414±0.0008)	(0.0441±0.0004)
GLY[e]	(0.0457±0.0008)	(0.0615±0.0004)	(0.0549±0.0003)
ETG[f]	(0.0371±0.0004)	(0.0491±0.0003)	(0.0426±0.0006)

a – 1,2-propanediol, b – diethylene glycol, c – triethylene glycol, d – 1,3-butanediol, e – glycerol, f – ethylene glycol

Table S3. Mole fraction solubility of ibuprofen (IP) in aqueous DES mixtures at various temperatures. In the first column, x^*_{DES} denotes the mole fraction of DES in solute-free mixtures with water. Standard deviation values are given in parentheses.

	temperature and DES system			
	**25°C**	**30°C**	**35°C**	**40°C**
x^*_{DES}	**choline chloride + TEG 1:2**			
0.1	(0.0211±0.0003)	(0.0257±0.0001)	(0.0318±0.0006)	(0.0386±0.0006)
0.2	(0.0641±0.0005)	(0.0783±0.0007)	(0.0973±0.0012)	(0.1210±0.0019)
0.3	(0.1227±0.0023)	(0.1520±0.0019)	(0.1876±0.0024)	(0.2338±0.0048)
0.4	(0.1961±0.0021)	(0.2424±0.0032)	(0.2953±0.0062)	(0.3695±0.005)
0.5	(0.2548±0.0063)	(0.3205±0.0083)	(0.3919±0.0029)	(0.4807±0.0064)
0.6	(0.2992±0.0052)	(0.3723±0.0052)	(0.4667±0.0108)	(0.5790±0.0052)
0.7	(0.3351±0.0045)	(0.4232±0.0081)	(0.5264±0.0138)	(0.6531±0.0080
0.8	(0.3673±0.0062)	(0.4557±0.0051)	(0.5712±0.0136)	(0.7095±0.0082)
0.9	(0.3754±0.0036)	(0.4619±0.0105)	(0.5742±0.0073)	(0.7138±0.0065)
1.0	(0.3528±0.0029)	(0.4366±0.0077)	(0.5483±0.0115)	(0.6784±0.0040)
x^*_{DES}	**betaine + TEG 1:2**			
0.1	(0.0396±0.0003)	(0.0475±0.0007)	(0.0561±0.0003)	(0.0688±0.0013)
0.2	(0.0911±0.0020)	(0.1077±0.0009)	(0.1301±0.0019)	(0.1547±0.0024)
0.3	(0.1423±0.0035)	(0.1670±0.0021)	(0.2005±0.0018)	(0.2400±0.0050)
0.4	(0.1950±0.0030)	(0.2288±0.0034)	(0.2772±0.0045)	(0.3302±0.0064)
0.5	(0.2459±0.0052)	(0.2949±0.0045)	(0.3502±0.0073)	(0.4150±0.0056)
0.6	(0.2941±0.0043)	(0.3535±0.0052)	(0.4213±0.0098)	(0.5066±0.0066)
0.7	(0.3409±0.0049)	(0.4057±0.0067)	(0.4779±0.0128)	(0.5883±0.0063)
0.8	(0.3800±0.0058)	(0.4493±0.0062)	(0.5335±0.0056)	(0.6375±0.0137)
0.9	(0.3582±0.0053)	(0.4279±0.0034)	(0.5040±0.0073)	(0.6012±0.0090)
1.0	(0.3209±0.0024)	(0.3830±0.0062)	(0.4548±0.0069)	(0.5444±0.0067)

Table S4. Mole fraction solubility of ketoprofen (KP) in aqueous DES mixtures at various temperatures. In the first column, x^*_{DES} denotes the mole fraction of DES in solute-free mixtures with water. Standard deviation values are given in parentheses.

	**temperature and DES system**			
	**25°C**	**30°C**	**35°C**	**40°C**
$\mathbf{x^*_{DES}}$	**choline chloride + TEG 1:2**			
0.1	(0.0056±0.0001)	(0.0076±0.0001)	(0.0102±0.0001)	(0.0136±0.0002)
0.2	(0.0147±0.0005)	(0.0195±0.0003)	(0.0265±0.0002)	(0.0356±0.0003)
0.3	(0.0248±0.0003)	(0.0331±0.0003)	(0.0446±0.0003)	(0.0602±0.0008)
0.4	(0.0338±0.0002)	(0.0457±0.0004)	(0.0610±0.0004)	(0.0826±0.0012)
0.5	(0.0419±0.0005)	(0.0560±0.0004)	(0.0757±0.0005)	(0.1047±0.0008)
0.6	(0.0503±0.0005)	(0.0680±0.0003)	(0.0927±0.0013)	(0.1254±0.0013)
0.7	(0.0583±0.0008)	(0.0784±0.0006)	(0.1065±0.0016)	(0.1449±0.0018)
0.8	(0.0656±0.0006)	(0.0885±0.0011)	(0.1198±0.0009)	(0.1613±0.0019)
0.9	(0.0684±0.0008)	(0.0928±0.0009)	(0.1251±0.0017)	(0.1700±0.0015)
1.0	(0.0670±0.0014)	(0.0906±0.0005)	(0.1219±0.0021)	(0.1639±0.0012)
$\mathbf{x^*_{DES}}$	**betaine + GLY 1:2**			
0.1	(0.0047±0.0001)	(0.0059±0.0001)	(0.0073±0.0001)	(0.0094±0.0001)
0.2	(0.0111±0.0001)	(0.0143±0.0002)	(0.0179±0.0002)	(0.0228±0.0002)
0.3	(0.0189±0.0003)	(0.0238±0.0003)	(0.0303±0.0003)	(0.0383±0.0003)
0.4	(0.0284±0.0003)	(0.0362±0.0004)	(0.0461±0.0003)	(0.0580±0.0005)
0.5	(0.0390±0.0002)	(0.0499±0.0004)	(0.0629±0.0006)	(0.0792±0.0007)
0.6	(0.0509±0.0004)	(0.0644±0.0009)	(0.0814±0.0009)	(0.1022±0.0010)
0.7	(0.0622±0.0005)	(0.0794±0.0013)	(0.1004±0.0015)	(0.1265±0.0014)
0.8	(0.0716±0.0009)	(0.0916±0.0009)	(0.1158±0.0012)	(0.1478±0.0012)
0.9	(0.0720±0.0005)	(0.0920±0.0008)	(0.1177±0.0008)	(0.1484±0.0009)
1.0	(0.0615±0.0004)	(0.0775±0.0012)	(0.0995±0.0011)	(0.1256±0.0011)
